# Facial Expression of TIPI Personality and CHMP-Tri Psychopathy Traits in Chimpanzees (*Pan troglodytes*): Evidence for Honest Signalling?

**DOI:** 10.1007/s12110-023-09462-2

**Published:** 2023-11-07

**Authors:** Lindsay Murray, Jade Goddard, David Gordon

**Affiliations:** 1 School of Psychology, University of Chester, Chester, UK; 2 School of Health, Science and Wellbeing, Staffordshire University, Stoke-On-Trent, UK

**Keywords:** Chimpanzee, Facial expression, Honest signalling, Personality, TIPI, CHMP-Tri

## Abstract

Honest signalling theory suggests that humans and chimpanzees can extract socially relevant information relating to personality from the faces of their conspecifics. Humans are also able to extract information from chimpanzees’ faces. Here, we examine whether personality characteristics of chimpanzees, including measures of psychopathy, can be discerned based purely on facial morphology in photographs. Twenty-one chimpanzees were given naïve and expert personality ratings on the Ten Item Personality Inventory (TIPI) and the Chimpanzee Triarchic Model of Psychopathy (CHMP-Tri) before and following behavioural observations. Characteristics relating to openness, conscientiousness, extraversion, and disinhibition could be distinguished from the faces of chimpanzees. Individuals higher on disinhibition have lower scores on conscientiousness and emotional stability and higher scores on extraversion, while those higher on meanness have lower conscientiousness and agreeableness. Facial expressions are linked to personality traits present in the TIPI and CHMP-Tri models: the Relaxed Face and the Grooming Face were displayed more by chimpanzees higher on agreeableness, whereas the Compressed Lips Face was observed more in those individuals higher on boldness, and the Full Open Grin was displayed more by chimpanzees higher on extraversion but lower on emotional stability and conscientiousness. Facial expressions were also found to be associated with particular behavioural contexts, namely the Grooming Face in affiliative contexts and the Relaxed and Relaxed Open Mouth Faces in neutral contexts. Dominant chimpanzees display higher levels of boldness and more Compressed Lips Faces, Relaxed Open Mouth Faces, and Grooming Faces than subordinate individuals. These findings support and extend evidence for an honest signalling system and a personality structure shared between humans and chimpanzees. Future research could further explore how personality is conveyed through the face, perhaps through more than just singular aspects of character, and maybe reflecting what chimpanzees themselves are able to do.

**Supplementary Information:**

The online version contains supplementary material available at 10.1007/s12110-023-09462-2.

Sharing 98% of their DNA with humans (Marks, 2002), chimpanzees (*Pan troglodytes*) are highly social primates living in large groups with complex relationships and interactions. Their communication through sophisticated vocal and gestural repertoires is well-represented in the literature (e.g., Goodall, 1986; Roberts et al., 2019); however, their facial expressions, less so, despite the existence of a dedicated facial analysis tool, the ChimpFACS (Parr et al., 2007). It is logical to presume that they would use information portrayed consistently through a range of facial expressions to help guide their interactions with conspecifics—for example, in joint attention activities and in dominance interactions (e.g., Flack et al., 2004). But, apart from intentional signalling, might the neutral facial expression of an individual convey important aspects of character? This study focuses on chimpanzee faces from several angles: first, we perform an initial test of the concept that characteristic traits of personality, including so-called psychopathic aspects of boldness, meanness, and disinhibition, are conveyed in the neutral faces of chimpanzees; second, we examine whether these traits are linked to specific facial expressions that may be intentionally displayed; and third, we measure whether these facial expressions are used in particular behavioural contexts. Next, we explore whether dominant and subordinate individuals differ in terms of personality and psychopathy traits and facial expressions. Finally, as a test of convergent validity when applying the Ten Item Personality Inventory (TIPI, Gosling et al., 2003) to chimpanzee personality for the first time, we explore whether specific TIPI scores correlate with scores on the Chimpanzee Triarchic Model of Psychopathy (CHMP-Tri, Latzman et al., 2016).

In order for a social hierarchical structure of dominance to form and function, animals must interact with each other in consistent ways (Funkhouser et al., 2017) through vocal and nonvocal communication and signalling systems, including body movements, vocalisations, pheromones, and facial expressions (Martin & Bateson, 2007; Maynard-Smith & Harper, 2003). A communication signal is a behavioural, physiological, and/or morphological characteristic that allows one animal to transmit information to another, facilitating the avoidance of harm and the acquisition of resources, including food, shelter, and mates (Bradbury & Vehrencamp, 1998). Signalling can aid inter- and intra-species identification (Laidre & Johnstone, 2013; Searcy & Nowicki, 2005) and is a key component of communication systems, involving an important relationship between signaller and receiver (Krebs & Dawkins, 1984; Maynard-Smith & Harper, 2003).

However, communication between animals may or may not be intentional or goal-directed (e.g., Roberts et al., 2019) and does not necessarily involve honest signals (Laidre & Johnstone, 2013). A signaller may, for example, intentionally convey false information to deceive a receiver for their own benefit (Dawkins & Krebs, 1978; Krebs & Dawkins, 1984; Searcy & Nowicki, 2005), although there can be penalties for such dishonest signalling—for example, if the dishonest signal invites aggression from conspecifics (Molles & Vehrencamp, 2001; Tibbetts & Izzo, 2010). In chimpanzees, while vocalisations can alert others that the signaller is in danger and in need of assistance, the callers may exaggerate their calls so that other individuals perceive the attack as more severe and are therefore more likely to help; this could arguably reflect a real threat or a manipulative desire for easy aid (Slocombe & Zuberbühler, 2007).

Chimpanzees also make extensive use of nonvocal signals; for example, they can differentiate between the facial expressions of conspecifics (Parr et al., 1998). Given the hierarchical nature of chimpanzee societies and the often aggressive nature of the displays that maintain or challenge social positions (Foster et al., 2008; Funkhouser et al., 2017; Muller & Wrangham, 2004), chimpanzees should be able to honestly signal their intent (Parr et al., 2005). We know, for example, that these apes make use of facial expressions, spatial cues, hand clapping and body postures to contextualise signals used during a play situation (Flack et al., 2004; Matsusaka, 2004) and that they can clearly distinguish between screams, play faces, and bared teeth displays (Parr et al., 1998). Such signals can also be given by individuals not engaged in play; for example, during juvenile play, a third-party adult may emit play signals to stop the play evolving into conflict (Flack et al., 2004).

This ability to signal intent in both dyadic and triadic relationships suggests that chimpanzees have a complex honest signalling system within which facial morphology is key (Kramer et al., 2011). In humans, approximately 60% of communication occurs through nonverbal cues, such as the face (Mehrabian, 1972), and research has consistently shown that humans make rapid decisions about individuals based on their faces alone (Bar et al., 2006). These decisions seem to be broadly accurate; participants have been able to accurately judge unknown individuals in the Big Five personality dimensions (Satchell et al., 2019) and correctly guess an individual’s sociosexual orientation (Antar & Stephen, 2021) based on a static photo. Amygdala activation in response to faces corresponds to negative personality traits such as psychopathy in the photographed individual (Gordon & Platek, 2009). The high phenotypic variation in human facial morphology is suggested to have evolved to facilitate social interaction (Sheehan & Nachman, 2014) and, given the advantages and disadvantages of different personality characteristics (Nettle, 2006), being able to honestly signal one’s unique personality within the face would potentially be of further benefit for efficient social coordination.

In terms of the morphological features that signal personality in humans, there is some evidence that recognition might be based on symmetry (Fink et al., 2005) or jaw line (Lv et al., 2022), but much research has focused on an individual’s facial width-to-height ratio (fWHR). This is because fWHR has been associated with socially salient characteristics such as dominant and psychopathic personality traits (Anderl et al., 2016; Carré & McCormick, 2008). Equally, fWHR has been shown to predict aggressive, deceptive, and combative behaviours (Carré & McCormick, 2008; Lefevre et al., 2013), but also prosocial and self-sacrificing behaviours (Stirrat & Perrett, 2012). Thus, fWHR could act as an honest signal of dominance since the cost (perceived untrustworthiness by, and increased antagonism from, conspecifics) might be offset by the signalled aggressiveness or willingness to escalate a conflict if one’s demands are not met (Stirrat & Perrett, 2010). This is analogous to other honest signals of formidability seen throughout the natural world (e.g., Tibbetts & Izzo, 2010). However, some recent work has suggested that the connection between fWHR and personality is weaker than previously assumed (Durkee & Ayers, 2021; Kordsmeyer et al., 2019), and there is debate as to whether a single or a few facial landmarks are likely to enable personality structure to be easily predicted.

In chimpanzees, the picture is somewhat different. In both species, sexual dimorphism in fWHR has only been found when skulls were measured (Weston et al., 2004, 2007) but not when measurements were taken from photos of faces (Kramer, 2017; Wilson et al., 2020). In contrast to humans, however, the relationship between dominance and fWHR is only present in one subspecies of chimpanzee (*Pan troglodytes verus*), and then only in adult females (Wilson et al., 2020). This is interesting when facial features of chimpanzees and humans are considered more broadly. In their study, Kramer and colleagues (2011) presented photographs of both human and chimpanzee faces to human participants and asked them to rate them for extraversion and dominance, dominance being part of the extraversion trait in humans (Goldberg, 1990). While human males who are more dominant have wider faces, as do the more dominant female chimpanzees mentioned above, it may be that dominance is conveyed in chimpanzees through other features, either singly or in combination. For example, pronounced brow ridges characteristic of the species, but more prominent in some individuals than others, may convey higher rank. For one thing, the brow ridge shades the eyes, and the ability to ‘read’ the eyes of a conspecific may be key in avoiding threat. Shadowed eyes, in addition to furrowed brows and a wider nose, were features manipulated in a study using morphed images to examine dominance in humans (Miao et al., 2022). It is likely that humans use a combination of facial expressions, body expressions, and the surrounding context rather than one individual signal from the face (Kret et al., 2013).

Nevertheless, while the exact features responsible for accurate personality assessment remain contentious, humans do seem able to assess other humans’ personality characteristics with broad accuracy. Given facial recognition is common across primates (Parr, 2011), it is logical to suggest that facial morphology is also involved in shared signalling, and that humans and chimpanzees may have similar systems underpinned by shared cognitive mechanisms. Thus, Kramer et al. (2011) proposed that humans can use facial cues from both other humans and chimpanzees to understand socially relevant behaviours, including dominance and personality characteristics such as extraversion. They concluded that chimpanzees and humans exhibit an honest signalling system due to their ability to extract information from the faces of both their conspecifics and other closely related species. A further study found that humans could accurately identify health information and personality characteristics, including emotional stability, extraversion, and agreeableness, from photographs of chimpanzee faces (Kramer & Ward, 2012).

The personality traits of chimpanzees have been measured using both bottom-up species-specific measures (Murray, 1998, 2011) and top-down human-derived measures relating to the Five Factor Model (FFM, Weiss et al., 2000). The applicability of this human measure to chimpanzees suggests similarities in personality structure between the species (Weiss et al., 2012). Collecting personality ratings of 100 chimpanzees from zoo employees and volunteers, King and Figueredo (1997) found that chimpanzees displayed the FFM traits of openness, conscientiousness, extraversion, agreeableness, and emotional stability, plus a sixth factor of dominance. Some of these personality factors are associated with a psychopathic personality in humans; for example, extraversion is linked to the boldness (dominance) aspect of psychopathy (Kramer et al., 2011), agreeableness is inversely linked to the meanness aspect (Latzman et al., 2016) and conscientiousness is inversely linked to disinhibition (Latzman et al., 2016). Chimpanzees may therefore have facial cues of personality which are identified and interpreted by their conspecifics (Kramer & Ward, 2012); these may facilitate selection for homophily in personality as a basis for friendships (Massen & Koski, 2014).

The FFM is measured in its shortest form by the Ten Item Personality Inventory (TIPI, Gosling et al., 2003). This scale, with proven reliability and validity in humans (Erhart et al., 2009; Woods & Hampson, 2005), has not yet been used extensively in other animals, but it was sensitive enough to uncover personality changes in elephants (*Elephus maximus*) in response to the death of herd members (Rutherford & Murray, 2021). Here we utilize this scale with chimpanzees because of the similarity in personality structure between humans and chimpanzees uncovered in studies using other measures based on the FFM (King & Figueredo, 1997; Weiss et al., 2012). Pederson et al. (2005) explored whether chimpanzee personality can predict behaviour using the six factors proposed by King and Figueredo (1997) and found that agonistic behaviours positively correlated with dominance and emotional stability and negatively correlated with dependability and agreeableness, whereas affiliative behaviours positively correlated with extraversion. Also using the FFM plus dominance, a further study uncovered links between dominance and high levels of boldness, low levels of agreeableness, and high levels of emotional stability (Dutton, 2008).

Our study builds on previous research (Kramer & Ward, 2012; Kramer et al., 2011) by an initial testing of the concept that characteristic traits of personality, including ‘psychopathic’ aspects of boldness, meanness, and disinhibition, are conveyed in the faces of chimpanzees. Some chimpanzee behaviours have been suggested to equate to elements of psychopathy—for example, agonism, increased sexual activity, courageous actions, and outbursts of anger (Lilienfeld et al., 1999). Latzman and colleagues (2016) developed the Chimpanzee Triarchic Model of Psychopathy (CHMP-Tri), which has proven reliability and validity; it splits psychopathy into three constructs: boldness, disinhibition, and meanness, each purporting to reflect a different aspect of psychopathy. Boldness reflects fearlessness and social dominance, disinhibition reflects a lack of behavioural restraint, and meanness reflects callous aggression, such as exploiting others for personal gain (Latzman et al., 2016). Both genes and environment contribute to psychopathic tendencies (Latzman et al., 2017). There are important links between the CHMP-Tri and TIPI scales; for example, definitions of disinhibition and conscientiousness are contrasting. Disinhibition encompasses the need for immediate gratification, symbolising a lack of behavioural restraint (Latzman et al., 2016) and conscientiousness is described as self-discipline and striving for achievement (Kurtz & Tiegreen, 2005). Thus, if a chimpanzee has high behavioural restraint, they should also have self-discipline; based on these definitions, a chimpanzee rated highly on disinhibition on the CHMP-Tri model should also be rated low for conscientiousness on the TIPI scale, resulting in a negative correlation. Conscientiousness may also negatively correlate with meanness; Paunonen and Jackson (1996) have proposed a moral component to conscientiousness, suggesting that conscientious individuals are dependable and reliable.

In addition to examining whether characteristic traits of personality could be determined from the neutral faces of chimpanzees, we examined whether these traits were linked to specific facial expressions displayed in particular behavioural contexts. Noninvasive observations were conducted at Chester Zoo, UK, recording information on facial expressions and the behavioural context in which they were displayed. The TIPI scale and CHMP-Tri model were used to conduct a ‘naïve’ personality rating at time 1 (T1) based on portraits of unfamiliar chimpanzees at Chester Zoo, and an ‘expert’ personality rating at time 2 (T2). Naïve ratings were completed by the rater (native English speaker) before conducting the observations, with no knowledge of the personality or behaviour of the chimpanzees. The expert ratings were then completed by the rater after the observations had ended, when familiarity with the chimpanzees had developed. The predictions for this study were that naïve ratings (T1) on the Ten Item Personality Inventory (TIPI) and/or Triarchic Model of Psychopathy (CHMP-Tri) will relate to the T2 ratings of the TIPI and CHMP-Tri (Prediction 1); that specific TIPI and/or CHMP-Tri scores will correlate with each other at T2 (P2); that rates of observed facial expressions will relate to TIPI and/or CHMP-Tri ratings at T2 (P3); that observed facial expressions will be associated with different behavioural contexts (agonistic, affiliative and/or neutral) (P4); and that dominant and subordinate individuals will differ in terms of personality and psychopathy traits and facial expressions (P5).

## Method

### Subjects and Study Site

Twenty-one chimpanzees (*Pan troglodytes*) at Chester Zoo (age range: 7 months to 53 years; 7 males and 14 females) were observed in this study. In recent years, a new female named Vila was introduced to the group and two new infants were born, the youngest being born in July 2019. A change in the dominance hierarchy was ongoing with a young adolescent, Carlos, challenging the alpha male, Dylan. The enclosure consisted of an inside (143 m × 12 m) and outside (2000 m^2^) area within which the chimpanzees could freely roam; however, the outside area was sometimes closed in response to weather due to the risk of injury. The enclosure included two climbing frames with ropes and wooden panels, one inside and one outside. The chimpanzees had an unlimited supply of water and random feeding times throughout the day. Ethics permission was obtained from the School of Psychology Ethics Committee at the University of Chester and permission to conduct the study was obtained from Chester Zoo.

### Procedure and Measures

#### Photographs

An initial naïve personality rating was completed using portraits of the chimpanzees (ESM). The portraits of the chimpanzees included one photograph of their face in a neutral expression. We tried to select photographs of individuals under conditions as comparable as possible. Most of them were of the chimpanzee in the outside enclosure, full-face facing front and with mouth closed. However, for a small minority of individuals, an alternative angle or open mouth was used because it included the clearest depiction of the eyes. The photographs were reviewed by an independent rater, who rated all of them as a neutral expression. No information on the name or personality of the chimpanzees was provided at this time in order to maintain a naïve personality rating based on facial morphology alone.

#### Ten Item Personality Inventory (TIPI) and Chimpanzee Triarchic Model of Psychopathy (CHMP-Tri) Scales

Each chimpanzee was rated using the Ten Item Personality Inventory (TIPI, Gosling et al., 2003; Table 1) and the Chimpanzee Triarchic Model of Psychopathy (CHMP-Tri, Latzman et al., 2016; Table 2) scales to obtain personality and psychopathy ratings at time 1 (T1). These personality ratings were repeated at the end of the study (T2). The TIPI scale uses 10 items to measure the Big Five personality dimensions represented in the Five Factor Model (FFM): extraversion, agreeableness, conscientiousness, emotional stability, and openness to experience (Gosling et al., 2003). Each item on the scale is rated using a 7-point Likert scale from 1 (disagree strongly) to 7 (agree strongly) (Gosling et al., 2003) and is scored independently. To calculate an individual’s score on each of the five factors, scores on the corresponding two items are added together and divided by two. However, because each factor has one ‘positive’ item and one ‘negative’ item, the latter needs to be reverse-scored to align the direction, thus yielding scores whose strength reflects the amount of that trait exhibited. As an example, if chimpanzee Boris scores 6 on ‘Extraverted, enthusiastic’ and 3 on ‘Reserved, quiet’, the 3 on ‘Reserved, quiet’ needs to be changed to a 5 (swapping positions on the 1–7 scale), then the 6 and the 5 are added together and divided by 2, meaning Boris’s extraversion score is 5.5.

**Table 1:** Ten item personality inventory (TIPI, Gosling et al., 2003)

1.	_____ Extraverted, enthusiastic
2.	_____ Critical, quarrelsome
3.	_____ Dependable, self-disciplined
4.	_____ Anxious, easily upset
5.	_____ Open to new experiences, complex
6.	_____ Reserved, quiet
7.	_____ Sympathetic, warm
8.	_____ Disorganized, careless
9.	_____ Calm, emotionally stable
10.	_____ Conventional, uncreative

TIPI scale scoring (“R” denotes reverse-scored items)

Extraversion: 1, 6R; Agreeableness: 2R, 7; Conscientiousness; 3, 8R; Emotional Stability: 4R, 9; Openness to Experiences: 5, 10R

**Table 2:** Chimpanzee Triarchic Model of Psychopathy (CHMP-Tri, Latzman et al., 2016)

Dimension and Item	Item Description
*Boldness*	
Dominant	Is able to displace, threaten, or take food from other chimpanzees. May express high status by decisively intervening in social interactions
Dependent (R)	Often relies on other chimpanzees for leadership, reassurance, touching, embracing and other forms of social support
Anxious (R)	Hesitant, indecisive, tentative, jittery
Fearful (R)	Reacts excessively to real or imagined threats by displaying behaviours such as screaming, grimacing, running away or other signs of anxiety or distress
Bold	Daring, not restrained or tentative. Not timid, shy, or coy
Timid(R)	Lacks confidence, is easily alarmed, and is hesitant to venture into new social or nonsocial situations
*Disinhibition*	
Impulsive	Often displays some spontaneous or sudden behaviour that could not have been anticipated
Inventive/Spontaneous	More likely than others to engage in novel behaviours. E.g., using new devices or materials in their enclosure
Irritable	Often seems in a bad mood or is impatient and easily provoked to anger or exasperation and consequent agonistic behaviour
Excitable	Easily aroused to an emotional state. Becomes highly aroused by situations that would cause less arousal in most chimpanzees
Calm (R)	Equable, restful. Reacts to others in an even, calm way. Is not easily disturbed or agitated
Socially inept/Intrusive	Acts inappropriately in a social setting
Jealous/Attention-seeking	Often troubled by others who are in a desirable or advantageous situation such as having food, a choice location, or access to social groups. May attempt to disrupt activities or make noise to get attention
*Meanness*	
Kind/Considerate (R)	Often consoles others in distress to provide reassurance
Affectionate/Friendly (R)	Seems to have a warm attachment or closeness with other chimpanzees. This may entail frequently grooming, touching, embracing, or lying next to others
Bullying	Overbearing and intimidating towards younger or lower-ranking chimpanzees
Manipulative	Is adept at forming social relationships for its own advantage, especially using alliances and friendships to increase its social standing. Chimpanzee seems able and willing to use others
Stingy	Is excessively desirous or covetous of food, favoured locations, or other resources in enclosure. Is unwilling to share these resources with others

(R) indicates a reverse-coded item.

The CHMP-Tri model characterizes psychopathy as the combination of three trait constructs: boldness, disinhibition, and meanness (Latzman et al., 2016), each comprising several items. Each dimensional construct reflects different aspects of psychopathy. Boldness reflects social efficacy, stress immunity, and low avoidance/fear behaviours, all of which can be combined to represent fear/fearlessness (Latzman et al., 2016). Fearlessness is a trait which can encompass social dominance, emotional resilience, and adaptive risk-taking. Disinhibition reflects the chimpanzees’ level of impulse control, lack of playfulness, and the need for immediate gratification; all of these factors symbolise a lack of behavioural restraint (Latzman et al., 2016). Lastly, meanness corresponds with callous aggression, including lack of empathy, lack of close attachments, rebelliousness, excitement-seeking, exploiting others, and empowerment through cruelty. Each assessment item had a behavioural description and was rated using a 7-point Likert scale ranging from 1 (Least descriptive of the chimpanzee) to 7 (Most descriptive of the chimpanzee) (Table 2). Some items are reverse-scored.

#### Behavioural Observations and Facial Expressions

**Fig. 1:**
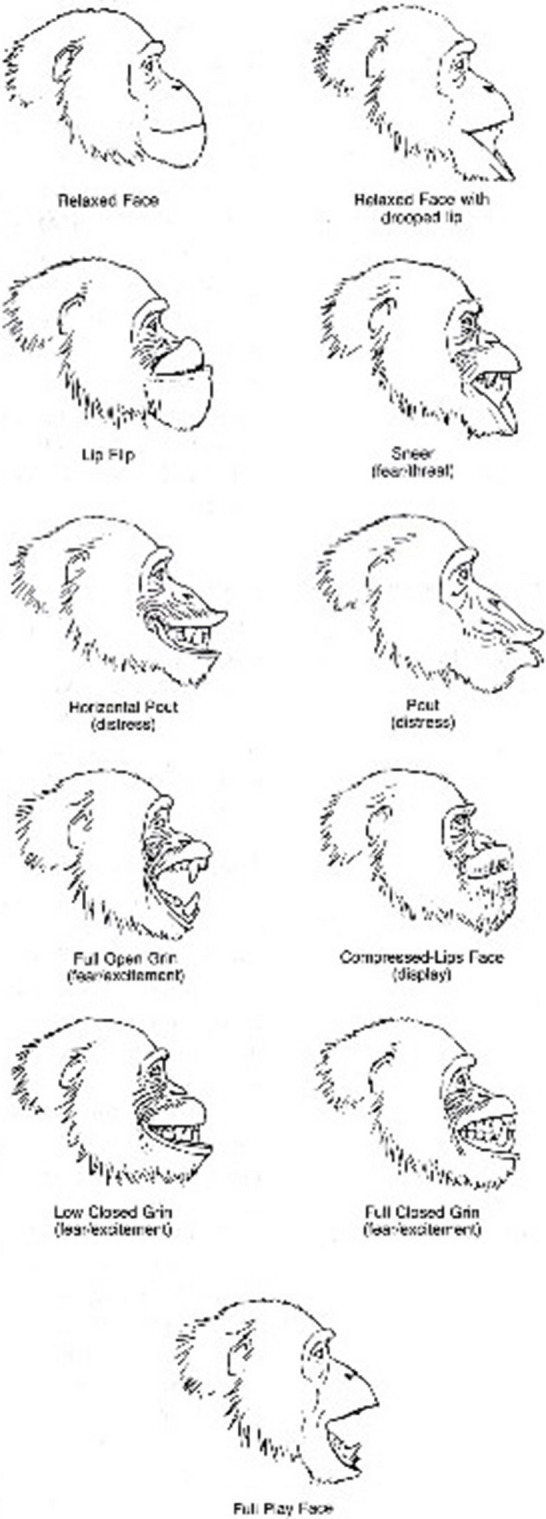
Ethogram of chimpanzee facial expressions (after D. Bygott, Fig. 6.1 in Goodall, 1986:120, used with permission)

**Fig. 2:**
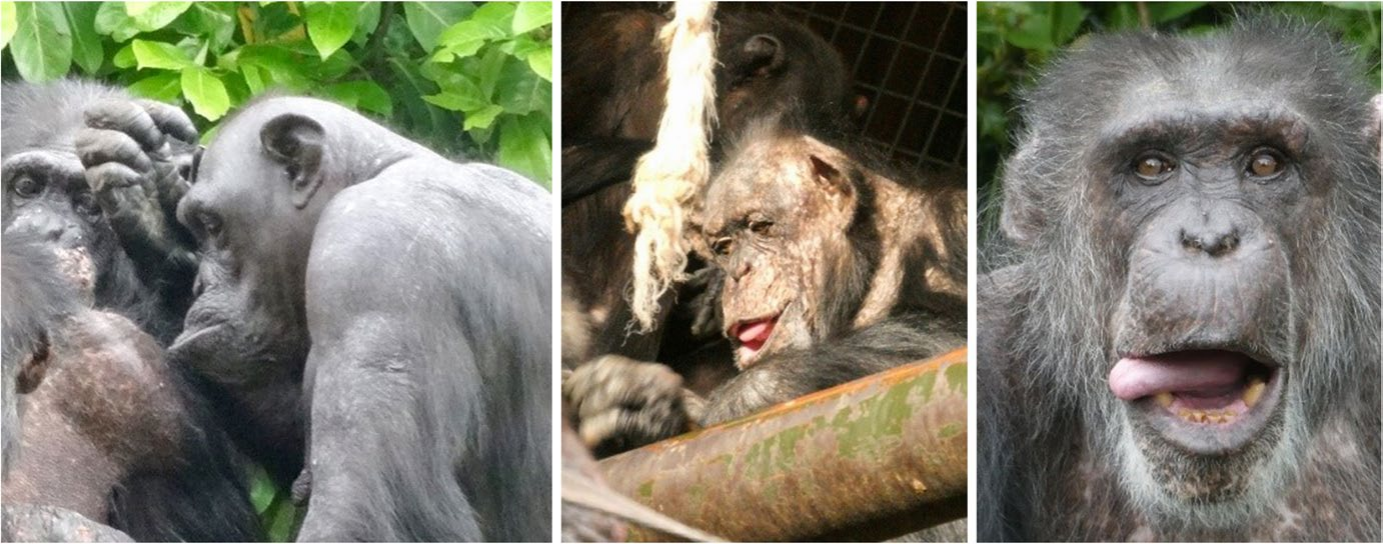
Illustration of the *Grooming Face* in adult female, Alice (left) and *Relaxed Open Mouth Tongue Out* in adults Sally (centre) and Boris (right), at Chester Zoo

**Fig. 3:**
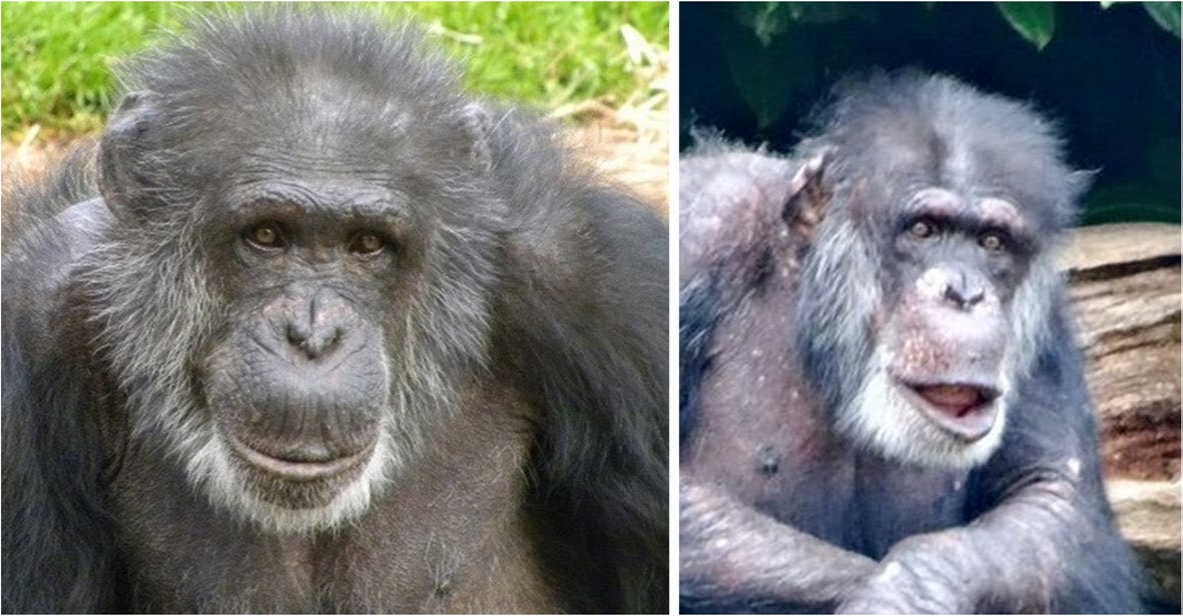
Examples of *Relaxed Face* (left) and *Relaxed Face with drooped lip* (coded and henceforth referred to as Relaxed Open Mouth) (right) in adult males, Boris and Friday, at Chester Zoo

An ethogram by Bygott (Fig. 6.1 in Goodall, 1986:120) of 11 illustrated facial expressions and their names was used to identify and code each facial expression displayed (Fig. 1). During the observations, two additional facial expressions were observed which were not portrayed in the Bygott ethogram. One we named the Grooming Face (GF), which is described as having the mouth directed towards the body of another chimpanzee, where the lips are either parted slightly (resembling the pout) or more often with the lower lip protruding and pointing slightly downward (thus somewhat resembling the drooped lip). The Relaxed Open Mouth Tongue Out (ROMTO) was also displayed, described as a relaxed face with open mouth, lip not drooped, and with the tongue sticking out. These can be seen in Fig. 2; other examples of real-life facial expressions are shown in Fig. 3.

Within approximately 35 h total, the observer sampled each focal individual for a minimum of six 15-min time periods (Altmann, 1974), within which the facial expression and the surrounding context were recorded as frequencies (Martin & Bateson, 2007). Behavioural contexts were coded as either affiliative (e.g., grooming/play), agonistic (e.g., aggression), or neutral (e.g., sitting still).

#### Design and Analysis

Noninvasive behavioural observations of facial expressions in different behavioural contexts were conducted at Chester Zoo from January 2020 until March 2020. Chimpanzees were rated before and following observations on the TIPI and CHMP-Tri scales. Inter-rater reliability was established via ICCs with another independent expert rater at the ‘expert’ rating stage before progressing with the analysis. Cronbach’s alpha was calculated for the CHMP-Tri scale but not for the TIPI since each factor only comprised two items. Because the sample size was relatively small, nonparametric tests were used (Shapiro–Wilk*, p* < .05). The naïve ratings at T1 (January 2020) for the TIPI scale and for the CHMP-Tri scale were correlated with the expert ratings at T2 (March 2020) (P1). Significant positive correlations would imply that it is possible to distinguish characteristic traits relating to personality in chimpanzees based on facial morphology. We also tested expected correlations between items on the TIPI and CHMP-Tri scores at T2 (P2).

Chimpanzees were also assigned a status of either dominant or subordinate based on independent expert judgements of the group. We decided to include this measure as another test of validity since ‘boldness’ in the CHMP-Tri model includes the personality aspect of social dominance. Dominance in chimpanzees has been measured both as a personality trait (King & Figueredo, 1997) and as a construct that reflects a group’s social hierarchy and the inter-individual relationships within it, these two measures being positively correlated (de Waal, 2000).

The data recorded were used to calculate the rate per hour of each facial expression for each chimpanzee. These rates per hour were analysed using Spearman’s correlation to test for relationships with TIPI ratings and CHMP-Tri ratings (P3). A chi-square test was also used to test for associations between the observed frequency of facial expressions across all chimpanzees with different behavioural contexts: affiliative, agonistic, and neutral (P4). Some of the facial expressions in the ethogram were rarely observed, so the top three most frequently observed facial expressions were used. Mann–Whitney *U *tests were used to assess differences between dominant and subordinate individuals in terms of personality, psychopathy, and facial expressions (P5).

## Results

Cronbach’s alpha was calculated for the CHMP-Tri scales but not for the TIPI since each factor only comprised two items. Boldness, disinhibition, and meanness all showed moderate internal consistencies: boldness (α = .50) and disinhibition (α = .63) were lower than expected based on Latzman et al.’s (2016) findings (boldness (α = .82) and disinhibition (α = .77)). However, the Cronbach’s alpha for meanness (α = .64) remained consistent with Latzman et al.’s (2016) original model (α = .67). Inter-rater reliability was established using ICCs between the rater and another independent expert rater at the T2 ‘expert’ rating stage before progressing with the analysis. These ICC (3, 1) reliabilities are shown in Table 3. The ICC (3, 1) reliabilities in Table 3 show substantial agreement between raters.

**Table 3:** Intraclass correlation coefficients (ICCs) for the TIPI and CHMP-Tri items

Item	ICC (3, 1)	95% CI	*p*
TIPI			
Openness	0.71	0.407–0.870	< .000
Conscientiousness	0.82	0.606–0.922	< .000
Extraversion	0.84	0.645–0.931	< .000
Agreeableness	0.85	0.659–0.935	< .000
Emotional Stability	0.63	0.288–0.833	.001
CHMP-Tri			
Boldness	0.42	− 0.015–0.722	.029
Disinhibition	0.82	0.601–0.925	< .000
Meanness	0.59	0.213–0.816	.002

### Prediction 1a: Naïve ratings (T1) of the Ten Item Personality Inventory (TIPI) will correlate with the expert (T2) ratings of the TIPI

Mean ratings across all chimpanzees were calculated for each item on the TIPI and the CHMP-Tri. From Table 4, it can be seen that the mean ratings at T1 and T2 are relatively similar for each of the five factors. T1 ratings for openness, conscientiousness, and extraversion are significantly positively correlated with the T2 ratings. T1 ratings for agreeableness and emotional stability did not significantly correlate with the T2 ratings.

**Table 4:** Mean ratings on TIPI at T1 and T2

TIPI trait	T1	T2	Spearman Correlation *r* (*N* = 21)
Openness	4.26	4.40	0.560 (*p* = .008)
Conscientiousness	4.02	4.31	0.603 (*p* = .004)
Extraversion	4.02	4.76	0.507 (*p* = .019)
Agreeableness	4.10	3.88	0.328 (*p* = .147)
Emotional Stability	4.10	3.67	0.058 (*p* = .801)

### Prediction 1b: Naïve ratings (T1) of the Triarchic Model of Psychopathy (CHMP-Tri) will correlate with the expert (T2) ratings of the CHMP-Tri

As can be seen in Table 5, the T1 ratings for disinhibition significantly correlated with the T2 ratings. For this reason, it may be possible to distinguish characteristic traits relating to disinhibition based on facial morphology but not to distinguish characteristic traits relating to boldness and meanness since the ratings for these constructs at T1 did not relate to the T2 ratings.

**Table 5:** Mean ratings on CHMP-Tri at T1 and T2

CHMP-Tri trait	T1	T2	Spearman Correlation* r* (*N* = 21)
Boldness (B)	4.58	4.87	0.436 (*p* = .055)
Disinhibition (D)	3.22	3.47	0.549 (*p* = .012)
Meanness (M)	3.63	3.18	0.344 (*p* = .137)

### Prediction 2: TIPI and CHMP-Tri scores will correlate with each other at T2

A Spearman’s correlation test between the TIPI scale and the CHMP-Tri model found that none of the five TIPI traits significantly correlated with boldness, suggesting that boldness is capturing something different from the personality factors. The expected correlations for disinhibition and meanness confirm convergent validity with the TIPI. Specifically, significant negative correlations were found between disinhibition and both conscientiousness and emotional stability, and a significant positive correlation was found between disinhibition and extraversion (Table 6). Conscientiousness also negatively correlates with meanness, as does agreeableness.

**Table 6:** Spearman correlations between TIPI scores and CHMP-Tri scores at T2

	CHMP-Tri trait		
TIPI trait	Boldness	Disinhibition	Meanness
Openness	− 0.343 (*p* = .138)	0.314 (*p* = .178)	− 0.160 (*p* = .500)
Conscientiousness	0.023 (*p* = .923)	− 0.690 (*p* = .001)	− 0.446 (*p* = .049)
Extraversion	0.197 (*p* = .404)	0.657 (*p* = .002)	0.296 (*p* = .204)
Agreeableness	− 0.117 (*p* = .624)	− 0.288 (*p* = .219)	− 0.818 (*p* < .001)
Emotional Stability	0.033 (*p* = .889)	− 0.631 (*p* = .003)	− 0.442 (*p* = .051)

### Prediction 3: Rates of observed facial expressions will relate to TIPI and/or CHMP-Tri ratings at T2

All facial expressions on the ethogram were observed at varying rates apart from the Lip Flip and Full Play Face, which were not recorded. In addition, two new facial expressions were observed: Grooming Face (GF) and Relaxed Open Mouth Tongue Out (ROMTO) (Fig. 2). Table 7 shows the mean rates per hour for facial expressions. As seen in Table 7, the ROM, RF, and GF have the highest mean value, indicating that they were the most observed facial expressions. In order to limit the number of multiple comparisons, we chose to focus our correlation analysis on specific facial expressions that might relate to particular personality traits. Focusing on representing variants of the most frequently observed expressions, we therefore selected the following to test: *Compressed Lips Face*—predicted to link with boldness ( +); *Full Open Grin*—predicted to link with conscientiousness ( −), extraversion ( −), emotional stability ( −), and boldness ( −); and *Relaxed Face*—predicted to link with extraversion ( +), agreeableness ( +), and boldness ( −). We had no reason to predict that the new face, *Relaxed Open Mouth Tongue Out*, would necessarily be associated with any particular traits; however, we did test whether the *Grooming Face* was linked with extraversion, agreeableness (as sociability proxies), meanness, and boldness (because bolder, more dominant individuals may engage in more grooming and meaner individuals, less so). Table 8 shows how the facial expressions correlated with personality traits.

**Table 7:** Mean (SD) rates per hour of observed facial expressions in chimpanzees (*N* = 21)

Face	Mean (SD)
Relaxed Face (RF)	**22.79 (18.07)**
Relaxed Open Mouth (ROM)	**16.35 (17.55)**
Grooming Face (GF)	**11.72 (18.00)**
Pout (PO)	**5.42 (5.61)**
Relaxed Open Mouth Tongue Out (ROMTO)	**2.33 (3.54)**
Full Open Grin (FOG)	**2.00 (3.30)**
Compressed Lips Face (CLF)	**1.58 (2.40)**
Full Closed Grin (FCG)	2.03(5.56)
Low Closed Grin (LCG)	0.65(1.72)
Horizontal Pout (H-PO)	0.28(1.31)
Sneer (S)	0.19(0.88)
Full Play Face (FPL)	0
Lip Flip (LF)	0

Bold denotes facial expressions exhibited by more than 8 individuals.

**Table 8:** Spearman correlations (*r*) between observed facial expressions, TIPI and CHMP-Tri ratings at T2 (*N* = 21)

	RF	GF	CLF	FOG
TIPI Trait				
Conscientiousness				− 0.453 (*p* = .039)
Extraversion	− 0.014 (*p* = .953)	0.008 (*p* = .973)		0.461 (*p* = .035)
Agreeableness	0.490 (*p* = .024)	0.448 (*p* = .042)		
Emotional Stability				− 0.520 *(p* = .016)
CHMP-Tri Trait				
Boldness	− 0.186 (*p* = .433)	0.251 (*p* = .285)	0.628 (*p* = .003)	0.270 *(p* = *.2*50)
Meanness		− 0.182 *(p* = .443)		

Bolder chimpanzees did indeed display more Compressed Lips Faces. Full Open Grins were linked with lower conscientiousness and emotional stability, as predicted, but with higher, rather than lower, extraversion, and there was no association with boldness. No associations were found between the Relaxed Face and extraversion and boldness, but the Relaxed Face was shown by more agreeable individuals, as predicted. Finally, the Grooming Face showed no associations with extraversion, boldness, or meanness but was displayed more by chimpanzees scoring higher on agreeableness.

### Prediction 4: Observed facial expressions will be associated with different behavioural contexts

The ROM, RF, and GF facial expressions with the highest mean rate per hour were used in a chi-square analysis. There were significant associations between the top three observed facial expressions (ROM, RF, and GF) and the behavioural contexts in which they were seen (χ^2^_4_ = 184.737, *p* < .001). As seen in Table 9, both the Relaxed Open Mouth and the Relaxed Face were observed most often in a neutral context, followed by an affiliative and then an agonistic context. They were observed more in a neutral context than expected, whereas in an affiliative and agonistic context, the observed count was lower than expected. The Grooming Face was observed most often in an affiliative context, followed by a neutral and then an agonistic context.

**Table 9:** Chi-square analysis testing an association between observed facial expressions (in contrast to expected counts) within each behavioural context

	Context		
Face	Affiliative	Agonistic	Neutral
Relaxed Open Mouth	20 (45.5)	4 (1.5)	95 (72.0)
Relaxed Face	37 (67.7)	1 (2.2)	139 (107.1)
Grooming Face	94 (37.8)	0 (1.3)	5 (59.9)

### Prediction 5: Dominant and subordinate individuals will differ in terms of personality and psychopathy traits and facial expressions

Table 10 shows the classification of each chimpanzee as either dominant or subordinate. Specifically, we tested whether boldness (CHMP-Tri) or extraversion (TIPI) differed according to dominance status. These two traits are arguably the characteristics in each of the personality models that would be expected to relate the most to dominance. In the development of the CHMP-Tri model, Latzman et al. (2016) state that boldness reflects the ability to display fearlessness and social dominance, which Lilienfeld et al. (1999) suggest is an important aspect of defining psychopathy in chimpanzees. For similar reasons, we tested whether more Compressed Lips Faces and Grooming Faces, and fewer Relaxed Open Mouth Faces, were shown by dominant individuals. Table 11 shows the results of the Mann–Whitney *U* tests for these variables, supporting the predictions that dominant chimpanzees scored higher on boldness and exhibited more Compressed Lips Faces and Grooming Faces. However, dominant chimpanzees actually exhibited more Relaxed Open Mouth faces too.

**Table 10:** Classification of chimpanzees as either dominant or subordinate

	Males	Females
Dominant	Boris	Sarah
	Dylan	Sally
	Carlos	Layla
	Eric	Alice
Subordinate	Wilson	Rosie
	Nicky	Farthing
	Friday	Mandy
		ZeeZee
		Vila
		Chrissie
		Patti
		Tina
		Stevie
		Annie

**Table 11:** Differences between high-ranking (*N* = 8) and low-ranking (*N* = 13) chimpanzees on predicted personality traits and facial expressions

	Mean Rank		Mann–Whitney Statistics
	Dominant	Subordinate	
Traits			
Boldness	13.81	8.29	*U* = 21.5, *p* = .040
Extraversion	14.00	9.15	*U* = 28.0, *p* = .079
Facial Expression			
Compressed Lips Face	15.00	8.54	*U* = 20.0, *p* = .008
Relaxed Open Mouth	14.69	8.73	*U* = 22.5, *p* = .031
Grooming Face	14.38	8.92	*U* = 25.0, *p* = .038

## Discussion

Supporting our first prediction, the findings suggest that it is possible to distinguish characteristic traits of personality relating to extraversion, openness, and conscientiousness from the faces of chimpanzees. Further, we present what we believe to be the first evidence that it is also possible to discern levels of disinhibition from chimpanzee faces. Supporting the second prediction, correlations were found between two of the CHMP-Tri traits and several TIPI traits: disinhibition with lower conscientiousness and emotional stability and higher extraversion; meanness with lower conscientiousness and agreeableness. Prediction 3 was also supported by several facial expressions relating to TIPI and CHMP-Tri ratings: both the Relaxed Face and the Grooming Face were displayed more by chimpanzees rated higher on agreeableness, the Compressed Lips Face was observed more on those individuals higher on boldness, and the Full Open Grin was displayed by those chimpanzees higher on extraversion, but lower on emotional stability and conscientiousness. Supporting the fourth prediction, facial expressions were found to be associated with particular contexts—namely, the Grooming Face in affiliative contexts and the Relaxed and Relaxed Open Mouth Faces in neutral contexts. Some support for the fifth prediction emerged with dominant chimpanzees displaying higher levels of boldness and more Compressed Lips Faces, Relaxed Open Mouth Faces, and Grooming Faces than subordinate individuals.

## Reading Personality from the Face

The significant positive correlations between naïve and expert ratings for openness, conscientiousness, and extraversion indicate that it is possible to distinguish these characteristic traits based on facial morphology alone. The finding for extraversion is consistent with previous research (Kramer & Ward, 2012; Kramer et al., 2011). Our study adds to this by providing the first evidence that characteristics relating to openness and conscientiousness can also be read from chimpanzees’ faces, as can the so-called psychopathic trait of disinhibition.

Therefore, the question that arises is what aspect of the face is leading to accurate personality judgements. In humans, research has often focused on the facial width-to-height ratio (fWHR) as this metric has been shown to be associated with psychopathic personality traits (Anderl et al., 2016) and predicts aggressive, deceptive, and combative behaviours (see Lefevre et al., 2013). Testosterone has been suggested as the mechanism explaining this association, with Lefevre et al. (2013) finding that, in men, fWHR was associated with both baseline testosterone levels and testosterone increase in response to a possible conflict. Since testosterone responds to competition, and higher levels are associated with higher aggression (Archer, 2006), human fWHR might honestly signal the likelihood that an individual will escalate a conflict, and this could be homologous in chimpanzees (Muller & Wrangham, 2004). However, recent studies in humans have failed to replicate the link between fWHR and testosterone (Bird et al., 2016; Kordsmeyer et al., 2019), suggesting that any honest signals embedded in facial features are not linked through its presence. Indeed, recent work has suggested that fWHR is a weaker predictor of the personality ratings given to faces than previously assumed (Durkee & Ayers, 2021).

This leaves the research area in a quandary since, although previous work on humans does indicate faces contain honest information about personality (Gordon & Platek, 2009; Lefevre et al., 2013; Santos et al., 2016; Satchell et al., 2019), exactly what features are being attended to is uncertain. It may be, as suggested by Kordsmeyer et al. (2019), that using only a few facial landmarks might not be valid for investigating the relationship between facial morphology and personality. A recent study by Kachur et al. (2020) made use of artificial neural networks to accurately predict Big Five personality in human faces, and such techniques might also be applicable to future nonhuman primate research.

## Intercorrelations between TIPI and CHMP-Tri

Some significant intercorrelations emerged between TIPI personality traits and the CHMP-Tri traits of disinhibition and meanness, but there were none with boldness. Negative correlations were found between disinhibition and both conscientiousness and emotional stability, while a positive correlation was found between disinhibition and extraversion. These findings provide support for the independence of the two scales but also for the validity of comparable definitions used in both the TIPI and CHMP-Tri models. Conscientiousness is also negatively correlated with meanness. In the CHMP-Tri model, factors in the meanness trait include manipulative behaviours, bullying, and being stingy. Furthermore, if a chimpanzee is stingy, they will not share their resources (Latzman et al., 2016). Therefore, they may not be dependable and/or reliable, suggesting the higher the meanness rating, the lower the conscientiousness rating, a pattern our findings support. Meanness and agreeableness also contrast in definition: an agreeable individual is trusting, cooperative, sympathetic, and generous (Kurtz & Tiegreen, 2005). If a chimpanzee is generous, they may share their resources with others, implying that a chimpanzee with an agreeable personality is less likely to be stingy or callously aggressive; hence, the negative correlation we uncovered between meanness and agreeableness is expected.

Lastly, according to Gosling et al. (2003), emotional stability involves the ability to remain calm, relaxed, and self-confident. As with disinhibition and conscientiousness, disinhibition and emotional stability are conflicting definitions; consequently, a higher disinhibition rating may result in a lower emotional stability rating, which is indeed what we found. In contrast to disinhibition and meanness, we suggest that the inclusion of boldness in the CHMP-Tri model may need reconsideration. Boldness in this model is described as reflecting fearlessness and social dominance, and we would agree with this description, but we argue that this does not necessarily constitute psychopathy. In humans, being bold may be linked to the confidence aspect of extraversion; in nonhuman animal personalities, the distinction between boldness and shyness was one of the earliest discoveries, having similarities to the approach/avoidance instinct (Reale et al., 2007). Our findings that boldness correlated with none of the other personality measures could attest to its primary focus on dominance, which plays a significant but separate role from personality and psychopathy in the lives of chimpanzees.

## Facial Expressions and Links to Personality

We identified two new facial expressions in this study: the Grooming Face, structurally resembling the Pout but with subtle differences and used solely in the grooming context, and the Relaxed Open Mouth with Tongue Out. Conversely, we did not observe the Full Play Face or the Lip Flip. Several of the facial expressions observed were associated with specific characteristic traits of personality. Two of the most frequently observed facial expressions—the Relaxed Face and the Grooming Face—were related to higher agreeableness. The Compressed Lips Face was related to higher boldness, and Full Open Grins were exhibited by chimpanzees rated higher on extraversion and lower on both emotional stability and conscientiousness. This supports and extends previous research which found that personality information can be perceived from the faces of chimpanzees (Kramer & Ward, 2012; Kramer et al., 2011).

## Facial Expressions Are Associated with Specific Behavioural Contexts

We found that the Relaxed Open Mouth (ROM) and Relaxed Face (RF) were associated with a neutral context and the Grooming Face (GF) was associated with an affiliative context, suggesting that facial signals in honest signalling systems may be associated with different contexts. For example, if a grooming signal is produced from the face by a signaller, the receiver may understand this signal and perform an affiliative behaviour, such as grooming, indicating that the context in which the signal is produced may be important in understanding and predicting the behaviour. The ROM has sometimes been linked to the play situation in chimpanzees, potentially because of a phylogenetic origin with human smiling and laughing (Waller & Dunbar, 2005), and in our study, we classified play as an affiliative behaviour. However, we found that the relaxed face occurred more often in neutral contexts, and this is consistent with earlier findings from matching tasks which showed that chimpanzees did not differentiate between relaxed lip faces and neutral faces (Parr et al., 1998).

The facial expression observed most often in an affiliative context was the Grooming Face (GF). This is unsurprising since grooming is a social behaviour which helps chimpanzees form and maintain social bonds and affiliative ties (Bonnie & de Waal, 2006. Affiliative ties form from the cooperation, coordination, and trust between grooming partners (Bonnie & de Waal, 2006). The ROM also significantly correlated with extraversion. As noted by Waller and Dunbar (2005), the ROM is associated with playfulness in chimpanzees, a factor which has been found to relate to extraversion (Pederson et al., 2005).

Goodall (1971) described the Full Open Grin (FOG) as a facial expression displaying excitement and/or fear, perhaps due to a high-ranking chimpanzee displaying nearby or if a chimpanzee is at risk of attack. In the present study, FOG correlated positively with extraversion and negatively with emotional stability and conscientiousness. Gosling et al. (2003) defined emotional stability as the ability to remain calm and relaxed without becoming stressed or anxious; therefore, since displaying a FOG is a sign of fear/excitement (Goodall, 1971), chimpanzees that demonstrate low fearfulness and high emotional stability should display fewer FOGs.

Furthermore, a FOG could be displayed more often by subordinate individuals (Goodall, 1971). Dutton (2008) found that dominant individuals score highly on extraversion. When this is taken into account, perhaps a FOG will be displayed less frequently by subordinate individuals who indicate low levels of extraversion. Therefore, a positive correlation between a FOG and extraversion challenges previous findings because it implies that more extraverted chimpanzees show more FOG expressions, a personality factor which has previously been associated with dominance in highly ranked chimpanzees. It could be that age plays a part and that youngsters rated higher on extraversion also get themselves into situations which result in the expression of a FOG; this would be worthy of further exploration. No significant correlations were found between the observed facial expressions and disinhibition and meanness.

## Differences between Dominant and Subordinate Chimpanzees in Personality and Facial Expressions

Kramer and colleagues (2011) found that humans could accurately distinguish personality traits relating to dominance from the faces of chimpanzees. Dominance has been found to be a factor of personality structures in humans and chimpanzees (King & Figueredo, 1997; Weiss et al., 2012). Here, we found that dominant chimpanzees were bolder and displayed more Compressed Lips Faces, but also more Relaxed Open Mouth faces and Grooming Faces. In the CHMP-Tri model, boldness reflects social efficacy, fearlessness, and social resilience (Kramer et al., 2012; Latzman et al., 2016), and it is likely that dominant individuals would engage in more grooming and hence exhibit more Grooming Faces, but the incidence of Relaxed Open Mouth faces is perhaps more surprising.

## Why Is the Reading of Chimpanzee Faces Important?

Chimpanzees are endangered, with their population decreasing every year (Maisels et al., 2016). From 1990 to 2014, Kühl et al. (2017) found an 80.2% decrease in the western chimpanzee population, with an annual decline of 6%. Researching captive chimpanzees (especially representatives of the western subspecies at Chester Zoo) can help develop greater understanding of how they behave in the wild, the best strategies for maintaining their wellbeing, and inform members of the public on the complexity of chimpanzee behavior and cognition, which can facilitate greater support for conservation efforts (Herrelko, 2011). Furthermore, understanding the influence of facial morphology, and the particular facial expressions utilised in communication systems, such as honest signalling, can help zoo staff determine the social hierarchy and increase their knowledge of how social systems work in a captive setting. For instance, if a facial expression is associated with a specific context, a staff member may be able to predict the action of the chimpanzee and intervene prior to any distress. Therefore, understanding honest signalling of personality and psychopathic traits in the faces of chimpanzees may help to maintain chimpanzee welfare in a captive setting.

A greater understanding of the individual character of chimpanzees can be facilitated from further exploration of some of the links we have identified between the Five Factor Model traits of personality and the CHMP-Tri traits of psychopathic personality. For example, individuals higher on conscientiousness are likely also to be lower on both meanness and disinhibition. Chimpanzees higher on disinhibition are more likely also to be higher on extraversion but lower on emotional stability. Just as humans make use of our implicit personality theory, including which aspects of personality tend to co-occur, it is possible that chimpanzees may also make use of facial conveyance of wider personality attributes. For example, they may extrapolate from a judgement about a given individual’s level of disinhibition to likelihood estimates of levels of wider emotional stability and extraversion. If this is the case, it would enable greater understanding and prediction of behaviour.

## Conclusion

In summary, our study provides a unique perspective on the inclusion of facial expressions in an honest signalling system of personality by incorporating psychopathy and dominance as factors which could contribute towards extracting socially relevant information from the face. We have added to the relatively limited literature in this area by showing that—in addition to extraversion—personality characteristics relating to openness, conscientiousness, and disinhibition can be discerned from the faces of chimpanzees. In addition, facial expressions relate to aspects of personality, including four of the personality dimensions in the Five Factor Model (all but openness) and two of the dimensions of psychopathic personality in the CHMP-Tri model (disinhibition and meanness). Facial expressions are also associated with specific behavioural contexts, and chimpanzees differing in dominance status exhibit both varied levels of personality traits and different facial expressions. Much research is now building on tools such as ChimpFACS (Parr et al., 2007) and using sophisticated AI technologies to rapidly identify individual chimpanzees from video footage (e.g., Schofield et al., 2019). We should now use deep convolutional neural network (CNN) approaches to further explore how facial expressions reflect personality traits and how they may act as indicators of welfare.

### Supplementary information

ESM 1  (PDF 956 KB)

## Data Availability

Data are available from the corresponding author upon request.
