# Supplementary material for: Facial Expression of TIPI Personality and CHMP-Tri Psychopathy Traits in Chimpanzees (Pan troglodytes): Evidence for Honest Signalling?
Source: Hum Nat. 2023 Nov 7;34(4):513–38. doi: 10.1007/s12110-023-09462-2 (PMC10739467; doi:10.1007/s12110-023-09462-2)
Supplement: Supplementary file 1 — (PDF 956 KB) [file 12110_2023_9462_MOESM1_ESM.pdf]

Electronic Supplementary Material for

Facial expression of TIPI personality and CHMP-Tri psychopathy traits in chimpanzees *(Pan troglodytes)*: Evidence for honest signaling?

Lindsay Murray[1], Jade Goddard[1] and David Gordon[1,2]
[1]School of Psychology, University of Chester, Chester, UK
[2] School of Health, Science and Wellbeing, Staffordshire University, Stoke-on-Trent, UK

Corresponding Author: Lindsay Murray l.murray@chester.ac.uk

Human Nature 34(4), 2023, https://doi.org/10.1007/s12110-023-09462-2

Photographs of the chimpanzees used for naïve ratings

TIPI Ratings	CHMP-Tri Ratings	Chimpanzee
		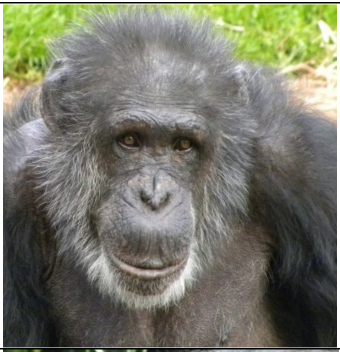
		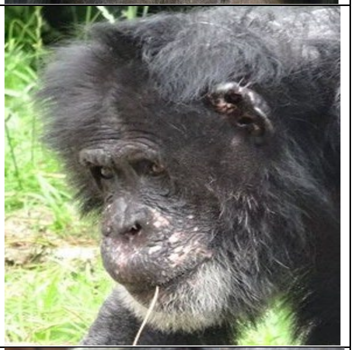
		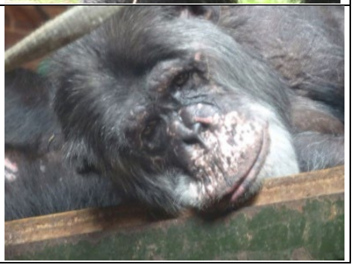

		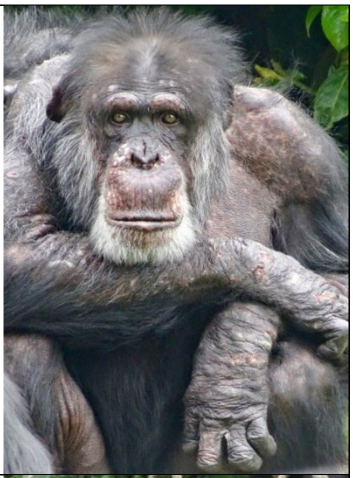
		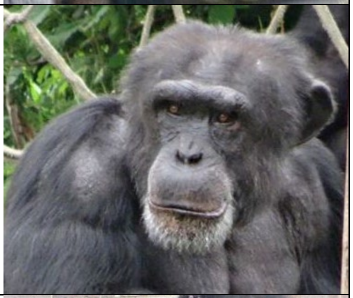
		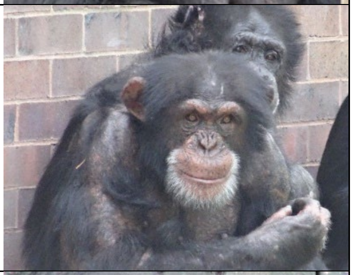
		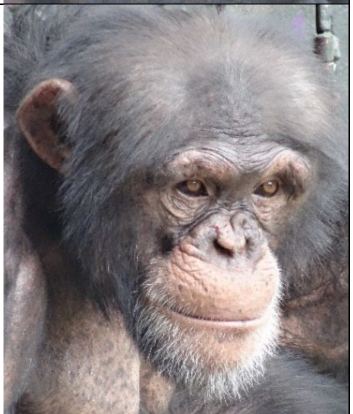
		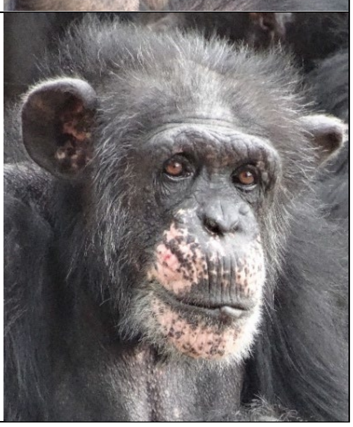

		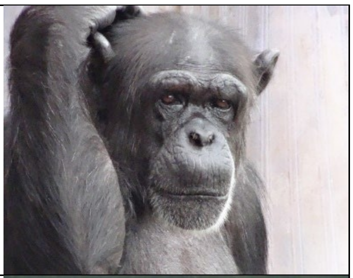
		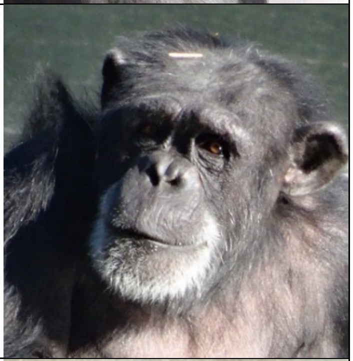
		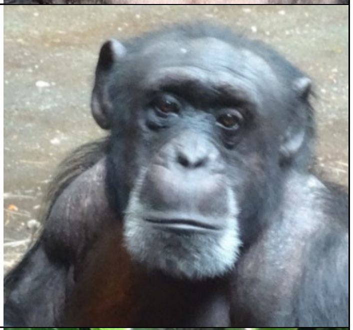
		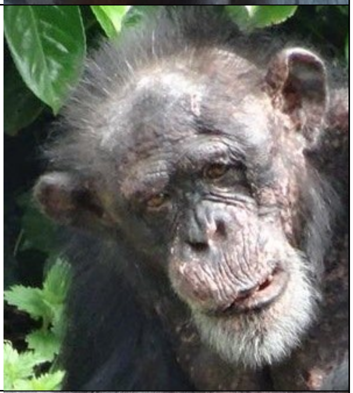
		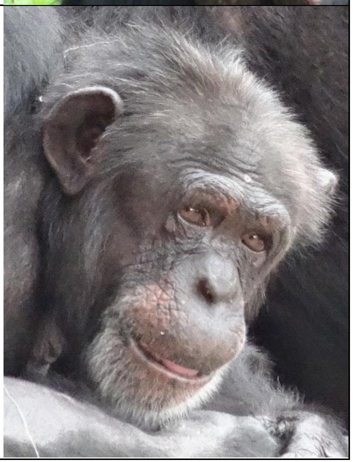

		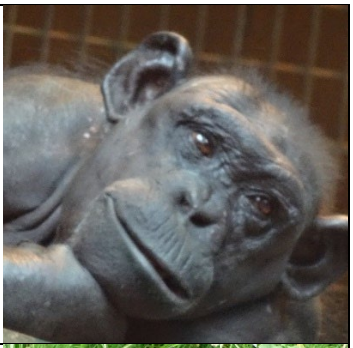
		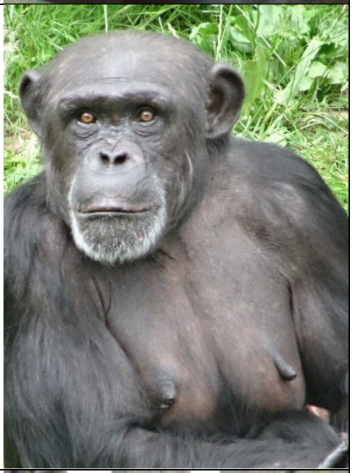
		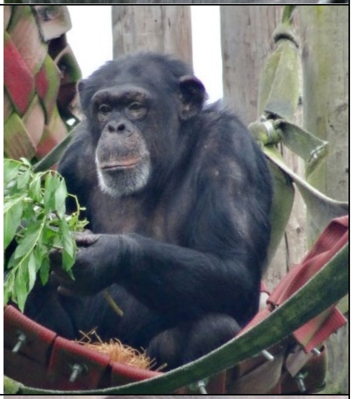
		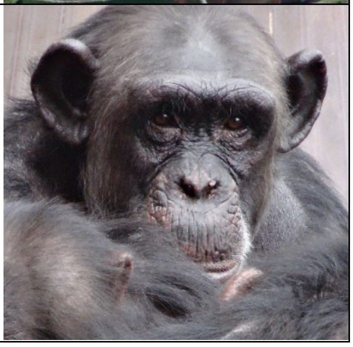

		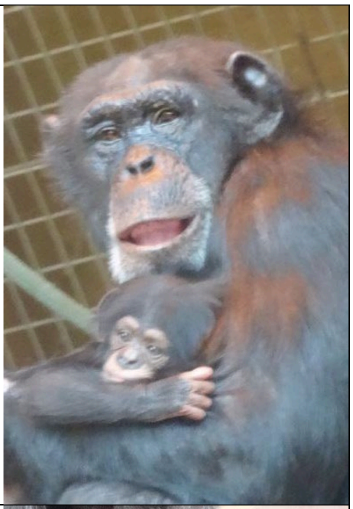
		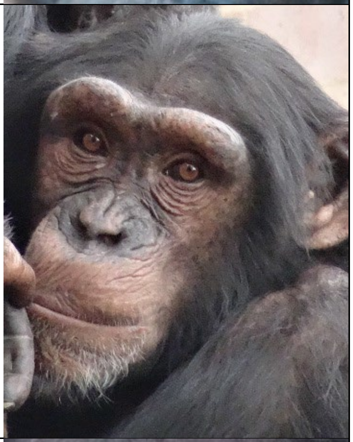
		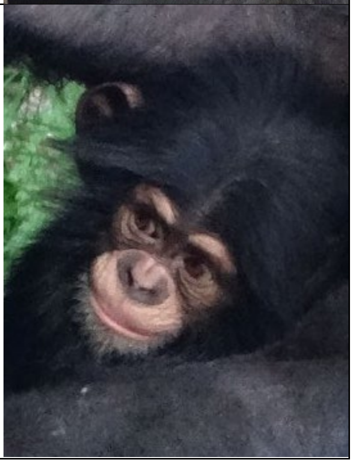

		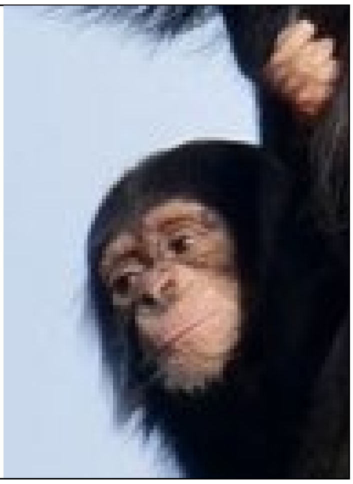
